# Oral vaccination of piglets against *Mycoplasma hyopneumoniae* using silica SBA-15 as an adjuvant effectively reduced consolidation lung lesions at slaughter

**DOI:** 10.1038/s41598-021-01883-2

**Published:** 2021-11-17

**Authors:** Marina L. Mechler-Dreibi, Henrique M. S. Almeida, Karina Sonalio, Mariela A. C. Martines, Fernando A. M. Petri, Beatriz B. Zambotti, Marcela M. Ferreira, Gabriel Y. Storino, Tereza S. Martins, Hélio J. Montassier, Osvaldo A. Sant’Anna, Márcia C. A. Fantini, Luís Guilherme de Oliveira

**Affiliations:** 1 School of Agricultural and Veterinarian Sciences, São Paulo State University (Unesp), Jaboticabal, Brazil; 2 Department of Chemistry, Federal University of São Paulo (UNIFESP), Diadema, SP Brazil; 3 Butantan Institute, São Paulo, Brazil; 4 Physics Institute, University of São Paulo (USP), São Paulo, Brazil

**Keywords:** Infectious diseases, Bacterial infection, Adjuvants

## Abstract

*Mycoplasma* (*M*.) *hyopneumoniae* is the main pathogen of porcine enzootic pneumonia (PEP). Its controlling is challenging, and requires alternative strategies. This study aimed to develop an oral vaccine against *M. hyopneumoniae* using a nanostructured mesoporous silica (SBA-15) as an adjuvant, and compare its effect with an intramuscular (IM) commercial vaccine (CV). Fifty 24 day-old *M*. *hyopneumoniae*-free piglets composed five equal groups for different immunization protocols, consisting of a CV and/or oral immunization (OI). Control piglets did not receive any form of immunization. All piglets were challenged with *M. hyopneumoniae* strain 232 on D49 by tracheal route. IgA antibody response in the respiratory tract, bacterial shedding and serum IgG were evaluated. The piglets were euthanized on 28 (D77) and 56 (D105) days post-infection. Lung lesions were macroscopically evaluated; lung fragments and bronchoalveolar fluid (BALF) were collected for estimation of bacterial loads by qPCR and/or histopathology examination. All immunization protocols induced reduction on *Mycoplasma*-like macroscopic lung lesions. IgA Ab responses anti-*M. hyopneumoniae*, the expression of IL-4 cytokine and a lower expression of IL-8 were induced by CV and OI vaccines, while IgG was induced only by CV. Oral immunization using silica as a carrier-adjuvant can be viable in controlling *M. hyopneumoniae* infection.

## Introduction

*Mycoplasma hyopneumoniae* (*M. hyopneumoniae*) is the main causative pathogen of porcine enzootic pneumonia (PEP), a chronic respiratory disease in pigs, and one of the main pathogens involved in the porcine respiratory disease complex (PRDC)^1^. The infections caused by this bacterium are highly prevalent worldwide and result in financial losses for the pig industry, mainly due to the costs of treatment and vaccination, decreased performance, and increased mortality from secondary infections^2^.

The microorganism's adhesion to the respiratory epithelium, the stimulation of a prolonged inflammatory reaction, the suppression and modulation of innate and adaptive immune responses favoring the pathogen are recognized as important steps in the colonization and infection by this microorganism. As a result, infected animals become more susceptible to infections by other respiratory pathogens^1^. As in other animals, most porcine pathogens cross mucous surfaces when ingested or inhaled, due to contamination of food, environment and fecal matter. Systemic vaccination generally promotes little stimulation of mucosal associated lymphoid tissue (MALT) and, therefore, the host immune system can only fight against the pathogen after its entering into the body^3,4^.

In the mucosal lymphoid tissues, mature T cells and B cells are stimulated by antigen and induce IgA antibody response. These cells migrate from the submucosal lymphoid tissue by the bloodstream to the lamina propria, where B cells differentiate into plasma cells secreting dimeric IgA antibodies. Many of these cells return to the original mucosal surface, but others can be found at different mucosal surfaces, so that oral immunization can lead to a migration of IgA precursor B cells to the bronchi, which subsequently secreted IgA antibodies in the bronchial mucosa^5^.

Previous studies with other pathogens have demonstrated the feasibility of using oral immunization as a strategy for inducing protective immunity in the swine reproductive tract, reinforcing the interconnection between different mucosal sites^6^. The secretory IgA (SIgA) specific antibodies have been considered as a crucial factor in protecting pigs against infection by *M. hyopneumoniae*^7^, while local humoral immunity seems to play an important role in this infection. SIgA is the main effector of respiratory tract mucosa immunity, which can form a protective barrier to eliminate respiratory invading pathogens and prevent infection and active colonization^8^. Since mucosal immunity has the potential to control pathogens at their portal of entry, it would be advantageous to develop vaccines that trigger a mucosal and systemic immune response rather than simply stimulating the systemic immune system^9^.

Promising *M. hyopneumoniae* bacterin formulations have been identified based on their capacity to induce strong innate immune responses^10^. Limitations in the use of adjuvants for vaccine formulation can be found in the literature, such as toxicity, the ability to lead to an immune response against the agent and not against the adjuvant, induction of adverse reactions, among other factors^11^. Since the silica has the characteristic of incorporating and releasing molecules^12^, the mesoporous silica particles have gained attention due to its potential role as adjuvants. Its toxicity to respiratory cells has been described^13,14^ and depends on the physicochemical properties of particles, size and concentration^15^. On the other hand, its use may enhance antigen-specific cellular immune responses in dendritic and Langerhans cells^15^. In addition, it is able to stimulate the immune system in a similar or superior way than other adjuvants, such as the Incomplete Freund's Adjuvant^16^. Moreover, an improvement in the recruitment of defense cells was observed, which led to an increase in phagocytosis and processing by the gut antigen-presenting cells^17–20^.

Based on the importance of developing oral vaccine adjuvants that are efficient in presenting antigens to the cells of mucosal lymphoid tissues (MALT), the objectives of this study were (i) to develop an oral vaccine specific to *M. hyopneumoniae* by encapsulating a blend of proteins of this bacterium into the silica (SBA-15); (ii) to stimulate the immune responses of the respiratory mucosa; (iii) to evaluate the efficacy of the protection induced by this vaccine against experimental infection with a virulent strain of *M. hyopneumoniae*, compared to a commercial inactivated vaccine.

## Results

### Mycoplasma-like macroscopic and microscopic lung lesion score at slaughter

At slaughter, gross lesions were mainly located in the apical and cardiac lung lobes, cranio-ventral portions of diaphragmatic lobe, and in portions of the intermediate lobe. All immunized groups showed lower lung lesion scores when compared to the control.

**Figure 1:**
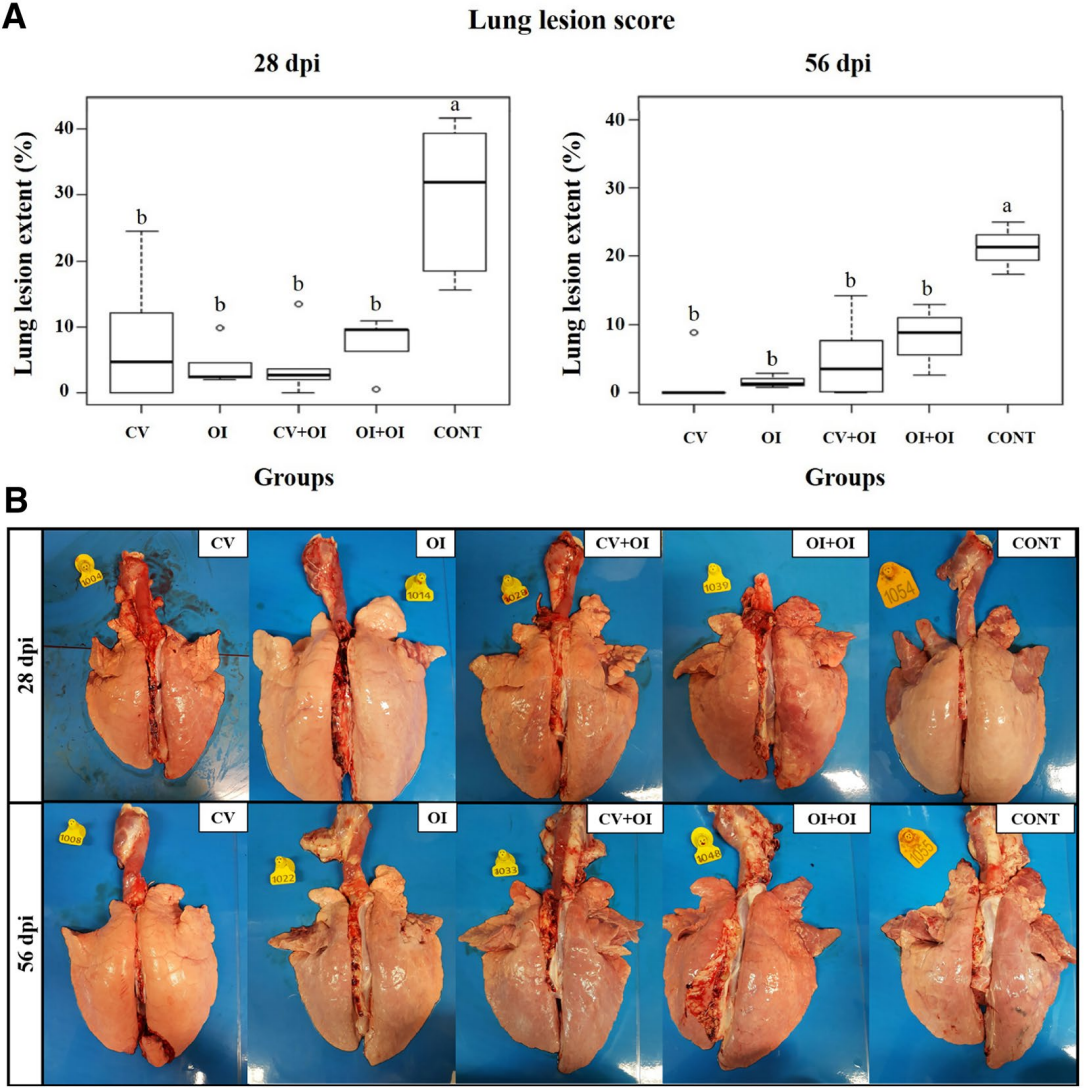
(**A**) Comparison of *Mycoplasma*-like macroscopic lung lesions extent observed at slaughter of piglets, 28- and 56-days post-infection with *M. hyopneumoniae*. Means followed by the same letter do not differ statistically from each other (Tukey test). (**B**) Representative photos of *Mycoplasma*-like macroscopic lung lesions extent observed at slaughter of piglets, 28- and 56-days post-infection with *M. hyopneumoniae*.

The medians of macroscopic lung lesion scores at the first slaughter (28dpi), followed by the lowest and higher scores observed in each group, were: CV-4.8% (0–24.5%), OI-2.5% (2–9.8%), CV + OI-2.7% (0–13.5%), OI + OI-9.6% (0.5–10.9%) and CONT-32.0% (15.6–41.7%). At the second slaughter (56 dpi), the values obtained were: CV-0.0% (0–8.8%), OI-1.3% (0.8–2.9%), CV + OI-3.5% (0–14.3%), OI + OI-8.8% (2.6–12.9%) and CONT-21.3% (17.4–24.9%). All immunized groups showed significant differences (Dunn test, p-value = 0.0229) in the total *Mycoplasma*-like lung lesion area when compared with the percentage obtained in the control. Significant differences were observed between the immunized groups and the control, but not between the immunized groups at both post-infection time-points (Fig. 1A). A difference in consolidation lung lesion score of 85% in CV, 92% in OI, 91% in CV + OI, 70% in OI + OI was observed in the first slaughter, while in the second slaughter the difference was of 100% in CV, 94% in OI, 83% in CV + OI, and 59% in OI + OI. Representative photos of consolidation lung lesion from each group are shown below (Fig. 1B).

**Figure 2:**
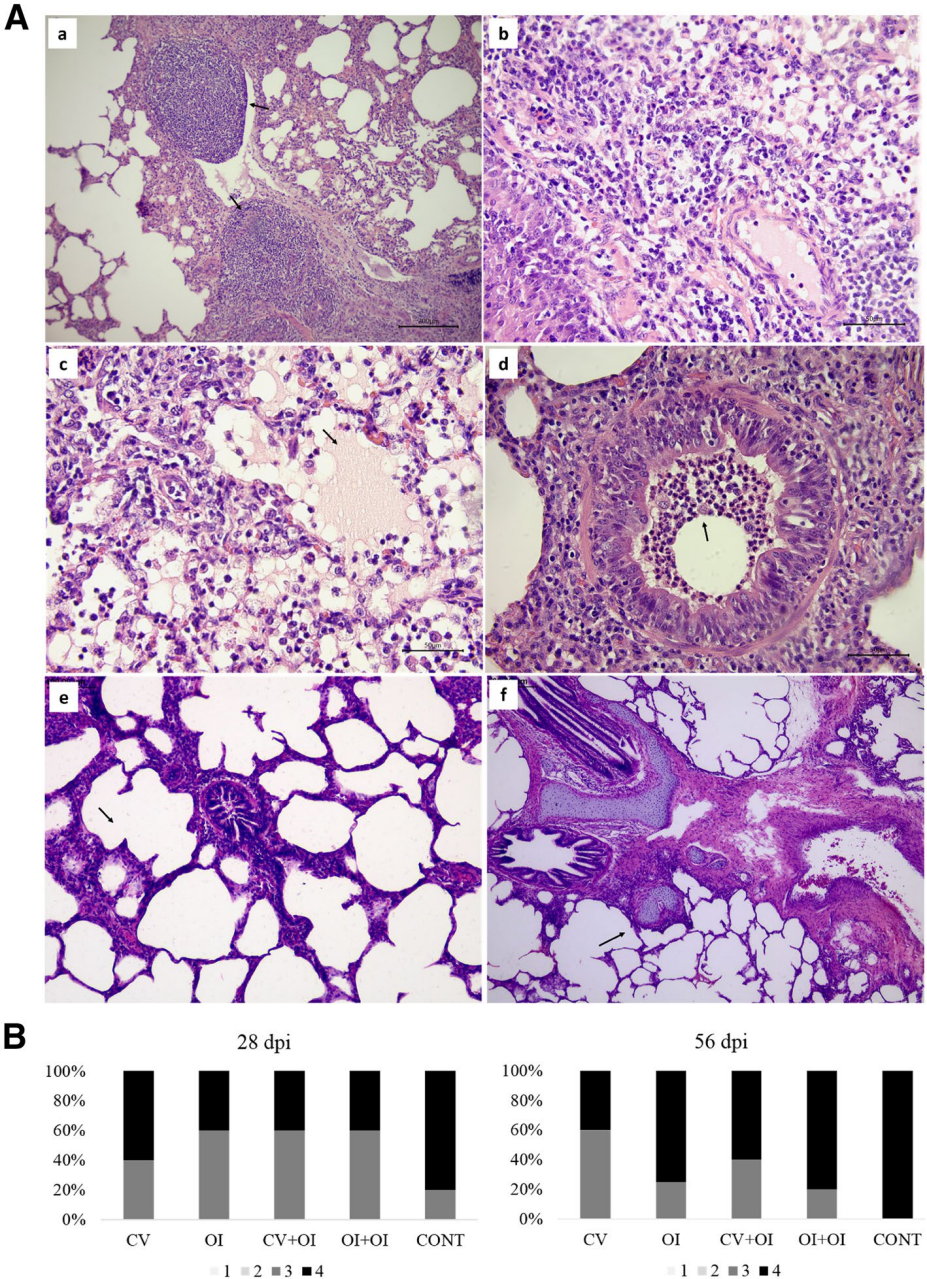
(**A**) Photomicrography of histological lung lesion characterized by (a) hyperplasia of lymphoid follicles (arrow) (100 ×); (b) inflammatory infiltrate predominantly compound by macrophages (400 ×); (c) amorphous and acidophilic material in addition to inflammatory infiltrate in the light of the alveoli (arrow) (100 ×); (d) inflammatory infiltrate in bronchioles (arrow) (400 ×); (e) light of the alveoli without noteworthy changes (arrow) (100 ×); (f) normal lymphoid follicle (arrow) (40 ×). (**B**) Percentage of animals showing different microscopic lung lesion score (0–4) according to each vaccination protocol. No significant differences were observed between groups.

Microscopically, all groups showed histological lesions score varying between 3 and 4, characterized as PEP-specific (Fig. 2A,B). No significant differences were found, neither between groups, nor between the two post-infection intervals. No significant correlations were observed between macro and microscopic analysis (Kruskall-Wallis, p-value = 0.341).

### Detection of IgA and IgG anti-*M. hyopneumoniae* antibodies in nasal swabs, serum and BALF samples

**Figure 3:**
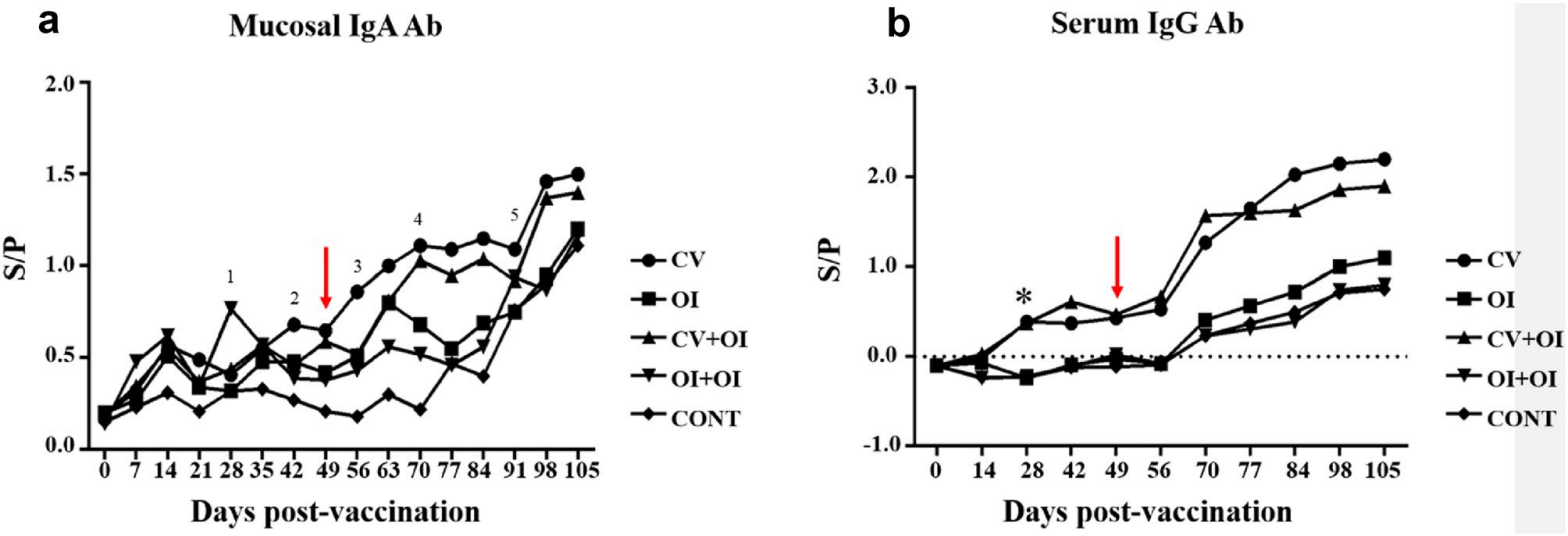
(**a**) Mucosal IgA antibody response related to different immunization protocols (D0) against *M. hyopneumoniae* along the experimental period. Dots represent the mean values of each group in each day of sampling. Positive S/P values > 0.4. (**b**) Serum IgG antibody response obtained in different immunization protocols (D0) against *M. hyopneumoniae* along experimental period. Piglets challenged with *M. hyopneumoniae* on D49 (red arrows). Dots represent the mean values of each group in each day of sampling. Positive S/P values > 0.3. Kruskall-Wallis test was used.

All immunized groups showed IgA Ab response in nasal swabs at 14 days post immunization, while the control group only became positive for IgA Abs 28 days post infection (Fig. 3a). The mean values with the respective standard deviation of each group and day of sampling, as well as statistical differences, are shown in Supplementary Table S3. On D28, OI + OI was statistically different from the other groups (for p-values, see Supplementary Table S5 online). The IgA responses of CV and OI at D42 and D49 were significantly different from the others. At D56, CV was statistically different from the other groups, while OI and CV + OI were statistically similar to each other and different from CONT. At D70, only CV and CV + OI IgA Ab responses were significantly higher than other groups, and OI was different from CONT. From D91 onwards, no differences were found between experimental groups. All piglets from immunized groups were IgA positive before challenge. When comparing the moments of slaughter (28 dpi and 56 dpi), significant differences (Two Sample t-test) were found for CV group (p = 0.045), CV + OI group (p = 0.028) and for CONT (p = 0.0001). No significant correlation was observed for any experimental group between the IgA Ab levels and the macroscopic lung lesion scores either at 28 or 56 dpi intervals.

The oral vaccine did not induce a serum IgG Ab response, whereas the commercial vaccine induced the highest levels of this antibody isotype (Fig. 3b). This result has to be further investigated by other experiments with larger concentration of antigens in SBA-15, providing no aggregation of proteins. It is important to point out that the concentration of antigen administered by the oral vaccine is much smaller than that the one used in the injected CV. The mean values with the respective standard deviation of each group and day of sampling, as well as statistical differences, are shown in Supplementary Table S4. All animals from the CV and CV + OI groups produced IgG anti-*M. hyopneumoniae* antibodies, and the Ab response started between D14 and D28, remaining with high levels until slaughter. From the moment that seroconversion was detected, the levels of IgG Ab were statistically similar for these two groups, but they differed from the others until the end of the experimental period (for p-values, see Supplementary Table S6 online). The animals from groups OI, OI + OI, and CONT seroconverted for IgG antibodies only four weeks after challenge with the pathogen (D70), and produced levels of IgG Ab statistically similar until the end of the experimental period. Comparing the differences between the moments of slaughter, significant differences (Two sample t-test) were found between the levels of IgG antibodies for CV (p = 0.008) and OI (p = 0.0003). No significant correlation was found for any experimental group between the IgG Ab levels and the macroscopic lung lesion scores, neither at 28 or 56 dpi intervals.

**Figure 4:**
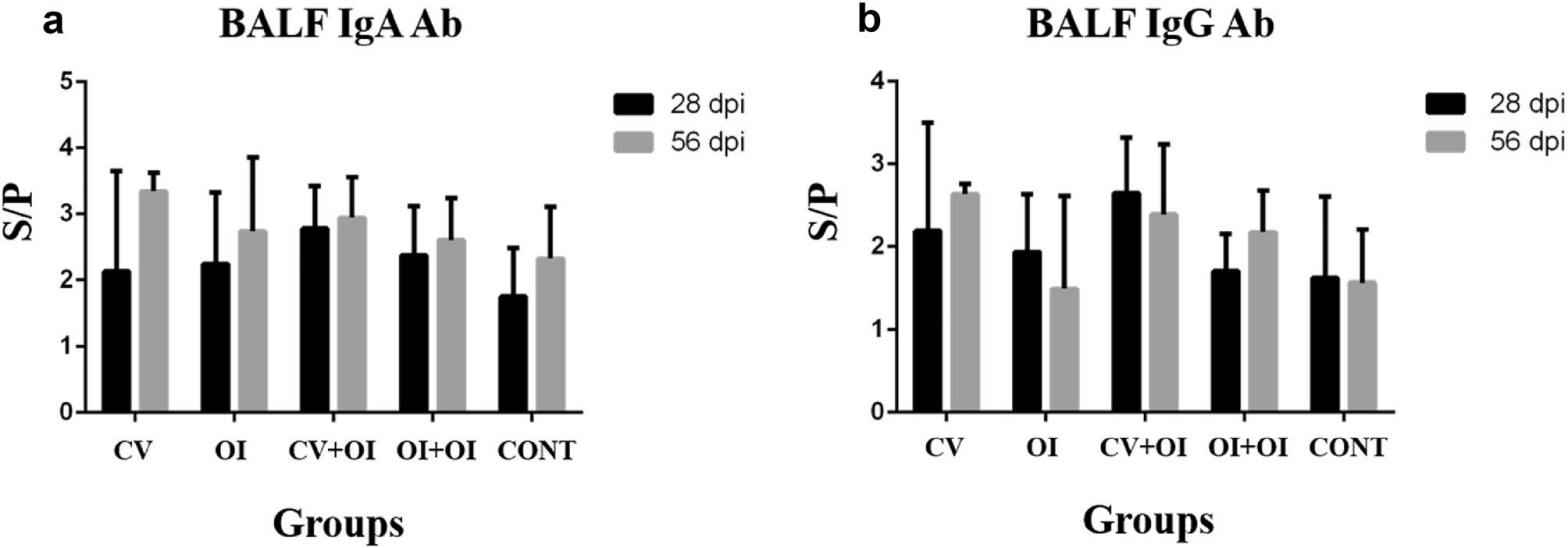
ELISA S/P (mean ± sd) results for BALF on 28- and 56 dpi regarding (**a**) IgA antibody response against *M. hyopneumoniae*; (**b**) IgG antibody response against *M. hyopneumoniae*. Positive S/P values > 0.4.

Regarding BALF, all groups showed positive responses to both IgA and IgG anti-*M. hyopneumoniae* Ab on 28 dpi and 56 dpi (Fig. 4). No statistical differences were found between groups for any of the evaluated immunoglobulins. All correlation analysis tested between variables are shown in Supplementary Table S7.

### Detection and quantification of p102 gene of *M. hyopneumoniae* by qPCR

The samples of nasal swabs tested by qPCR indicated that at 7th dpi there were piglets already shedding the pathogen in all experimental groups in different proportions, and by the end of the evaluated period, most of the piglets from all groups were shedding intermittently *M. hyopneumoniae* (Table 1). No significant differences were found between groups along the experiment.

**Table 1:** Number of piglets shedding *M. hyopneumoniae* in nasal swabs collected from the 7th to the 56th day post-infection with the pathogen, detected by qPCR.

	7 dpi	14 dpi	21 dpi	28 dpi	35 dpi	42 dpi	49 dpi	56 dpi
CV	2/10	7/10	7/10	4/5	5/5	2/5	4/5	5/5
OI	4/10	9/10	4/10	2/5	3/5	3/5	4/5	5/5
CV + OI	3/10	5/10	5/10	0/5	4/5	3/5	4/5	5/5
OI + OI	4/10	4/10	7/10	3/5	4/5	5/5	4/5	5/5
CONT	5/10	6/10	5/10	2/5	5/5	5/5	5/5	5/5

*Mycoplasma hyopneumoniae* was detected and quantified in lungs and BALF of all piglets from all groups, and the respective estimate loads and standard deviation are shown in Table 2. No statistical differences (Two Sample t-test) were found for all groups in both sampled time-points (28 and 56 dpi). Considering the correlation between macroscopic lung lesion and *M. hyopneumoniae* estimate quantification in lungs, a lower quantification was found to be strongly correlated with a reduced macroscopic lung lesion in CV + OI at 28 dpi (Spearman’s correlation coefficient R = 1; p = 0.01667), with a trend on group CV (p = 0.0538, R = 0.87). A lower quantification was also correlated with a reduced *M. hyopneumoniae* quantification in the BALF (28dpi) of CV (R = 0.99, p = 0.0051), OI (R = 0.97, p = 0.0299), CV + OI (R = 0.96, p = 0.03), and on 56 dpi in OI + OI (R = 0.99, p = 0.0004).

**Table 2:** Results of *p102* gene estimate quantification by qPCR in lungs and BALF of piglets from all experimental groups at the 28th and 56th days post-infection.

*p102* gene estimate quantification by qPCR				
Groups	Lungs (± sd) 28 dpi	Lungs (± sd) 56 dpi	BALF (± sd) 28 dpi	BALF (± sd) 56 dpi
CV	3.1 × 10^4^ (± 3.3 × 10^4^)	2.3 × 10^4^ (± 4.8 × 10^4^)	4.3 × 10^5^ (± 3.0 × 10^5^)	3.2 × 10^5^ (± 2.7 × 10^5^)
OI	2.5 × 10^4^ (± 2.8 × 10^4^)	4.4 × 10^4^ (± 3.6 × 10^4^)	2.1 × 10^6^ (± 1.9 × 10^5^)	3.4 × 10^5^ (± 3.5 × 10^5^)
CV + OI	1.3 × 10^4^ (± 1.7 × 10^4^)	3.0 × 10^4^ (± 3.6 × 10^4^)	1.5 × 10^6^ (± 1.5 × 10^6^)	3.0 × 10^5^ (± 3.4 × 10^5^)
OI + OI	1.3 × 10^5^ (± 1.6 × 10^5^)	4.7 × 10^4^ (± 5.8 × 10^4^)	2.3 × 10^6^ (± 1.5 × 10^6^)	9.5 × 10^5^ (± 1.3 × 10^6^)
CONT	1.0 × 10^5^ (± 7.4 × 10^4^)	1.7 × 10^5^ (± 2.0 × 10^5^)	1.0 × 10^6^ (± 7.0 × 10^5^)	2.2 × 10^6^ (± 2.4 × 10^6^)

No statistical differences were observed between groups or time-points.

### Cytokine coding gene’s expression and correlation with antibody levels and bacterial loads

**Figure 5:**
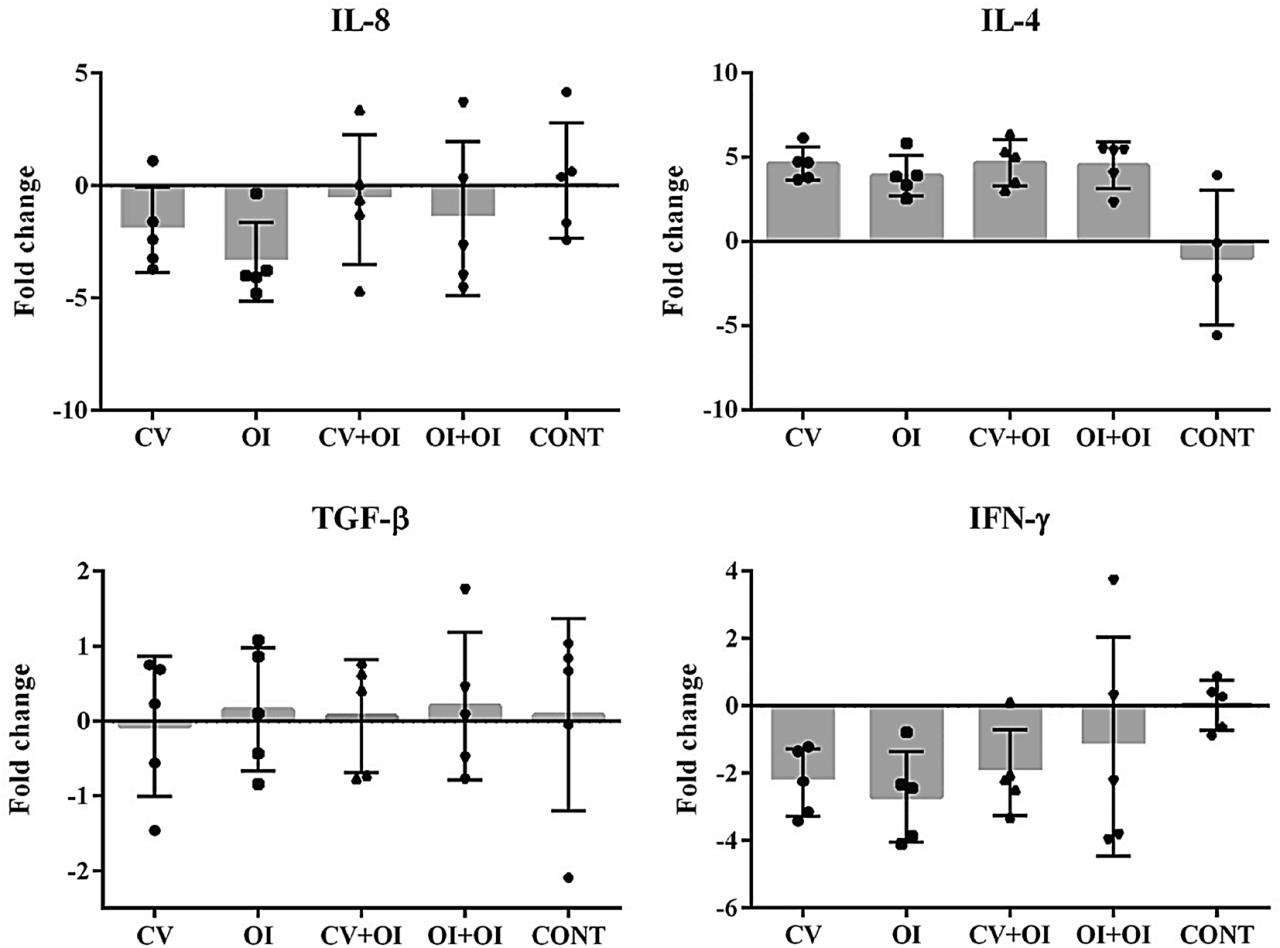
Bar graphs representing the fold change of cytokine gene expression (Fold Change mean ± sd) in lung lesion samples of five pigs per group, 28 days after experimental infection with *M. hyopneumoniae* strain 232, previously submitted to different immunization protocols. Target gene expression was normalized based on *rpl-4* gene expression. No statistical differences were found between groups (Kruskall-Wallis test).

Interleukin 8 was downregulated in immunized groups at 28 dpi compared to the control group, with a slight non-significant difference in the CV + OI group (Kruskall-Wallis test). Similarly, IFN-γ was less expressed in the immunized groups when compared to the control group, especially in CV, OI and CV + OI, with no significant differences between them. A significant and negative correlation was found between IFN-γ and IgA Ab response at 28 dpi (Pearson’s correlation coefficient R = − 0.43; p-value = 0.028). On the other hand, IL-4 expression was up regulated in all immunized groups, while it was less expressed in the control group. TGF-β expression was reported in all groups, with non-statistical differences between them (Fig. 5). TGF-β expression was positively correlated with IgA Ab response in the upper respiratory tract (nasal swabs) of piglets (Pearson’s correlation coefficient R = 0.95; p-value = 0.0401). All correlation analyses between variables are shown in Supplementary Table S7.

## Discussion

Oral vaccines are desirable in pig industry due to the relative ease of administration for large populations. In the present study, an oral vaccine for weaned piglets was developed by using the SBA-15 nanostructured mesoporous silica as a vehicle for a blend of *M. hyopneumoniae* proteins. Four immunization protocols were tested, followed by an experimental inoculation of *M. hyopneumoniae* strain 232, to evaluate the action of the oral and the intramuscular vaccines, alone or combined, and the results were compared with a non-immunized group. According to the manufacturer (MSD Animal Health), the commercial vaccine contains the proprietary dual adjuvant EMUNADE, capable of producing a rapid and prolonged immune response due to the aluminum hydroxide and an oil emulsion, respectively. As we have no information regarding the bacterial load present in M + PAC® and different routes of administration were performed, comparing its efficacy to the experimental oral vaccine is challenging. However, this IM vaccine was found to be an interesting option as a basis for comparison since it has been used worldwide and provided satisfactory results. In addition, most works with oral or aerosol vaccination against *M. hyopneumoniae* in piglets did not perform experimental challenge with this pathogen, which can limit the comparison of our results, such as bacterial shedding and quantification in the lugs, with other vaccines.

All immunization protocols showed reduced *Mycoplasma*-like lung lesion, and all groups presented a significant difference in lung lesion score when compared with the control group, being the highest differences observed in the medians of OI and CV + OI in the first slaughter (reduction of 92% and 91% of consolidated area, respectively), and in CV and OI (reduction of 100% and 94%, respectively) at the second slaughter. The oral and the commercial vaccines offered effective protection individually, but no combination effect with the two immunization ways were observed. These are promising findings, especially when compared to previous works in which no significant differences in lung lesions were found between vaccinated and non-vaccinated groups on the field with natural infection^21^, and with homologous and heterologous vaccines followed by experimental infection with *M. hyopneumoniae*^22^. On the other hand, microscopic lung lesions were statistically similar in all groups, vaccinated or not, with histological findings characteristics of *M. hyopneumoniae* infection, as also observed by Almeida^23^, after experimental infection using the same *M. hyopneumoniae* 232 virulent strain. An oral vaccine with recombinant *E. rhusiopathiae* strain expressing the *M. hyopneumoniae* P97 protein reduced the severity of pneumonic lung lesions caused by *M. hyopneumoniae* infection^24^. Similarly, a number of *M. hyopneumoniae* commercial inactivated vaccines administered by IM route provided such type of protection^25–27^.

*Mycoplasma hyopneumoniae* induces innate and adaptive immune responses, which is able to prevent significant systemic spread of the organisms. However, the immune system is unable to rapidly clear pulmonary airways infection, resulting in a prolonged localized inflammatory and cellular immune response, responsible for the majority of gross and microscopic lesions^1^. The vaccines and vaccination programs tested in the current study were able to induce IgG (commercial vaccine only) and IgA (commercial and experimental oral vaccines) antibodies against *M. hyopneumoniae*. Only the groups that received IM vaccination were able to produce serum IgG Ab detectable by ELISA test. It is well known that systemic anti-*M. hyopneumoniae* antibodies are considered to play a minor role in protection against PEP^28,29^. On the contrary, it is believed that specific locally secreted IgA may play a protective role by preventing the adhesion of the pathogen to the ciliate epithelium^30^. Independently on the presence of serum IgG Ab, all vaccinated animals showed a significant reduction of consolidation lung lesions, which allows to infer that mucosal IgA Ab probably participates in the protection and prevention against *M. hyopneumoniae* invasion and adherence, corroborating Martelli^30^. In this study, both oral and IM vaccines were capable of inducing IgA Ab production, which was found in the respiratory tract, as all immunized piglets were positive for this Ab at 14 days post-vaccination. Feng^31^ also observed the presence of IgA Ab in nasal cavities 14 days after administration of an aerosol vaccine against *M. hyopneumoniae,* although no challenge was performed by these authors.

Our results differ from previous ones that only detected significant antibody immune response after challenge infection^24^, and from studies using commercial vaccine which were not able to induce IgA Ab responses^31,32^. We have found that vaccinated pigs presented significantly higher antibody levels than the non-vaccinated ones, which may indicate a memory humoral immune response, either for IgA or IgG Abs. It is noteworthy that antibodies induced by IM vaccination may occur between 3 to 4 weeks after vaccination^33^, while seroconversion in natural *M. hyopneumoniae* infected pigs usually occurs around 8–24 weeks of age^34–36^. In the current study, seroconversion in the challenged piglets occurred 3 weeks post-infection. This early immune response, when compared to naturally infected ones, may be due to the differences in experimental and natural bacterial loads. The infecting dose under field conditions is expected to be lower, especially in farms with good management and biosecurity practices^37^. Determining the ideal time point to experimentally infect the piglets after vaccination is challenging, once vaccination and infections occur dynamically in field conditions. Furthermore, the primary or boosted immune response must be considered in the dynamics of *M. hyopneumoniae* infection. In the present study, the infectious challenge occurred two weeks after the booster vaccination, and the piglets from CV + OI and OI + OI could be still experiencing the effects of the immunization at that time. For this reason, further studies evaluating different moments of immunization in the field are necessary to a better understanding of this situation.

When considering the Ab immune response in BALF at slaughter (28 dpi and 56 dpi), all groups showed high S/P values for both IgA and IgG Ab, despite the statistically non-significance. As these samples were taken only at slaughter, it was not possible to determine the contribution of each vaccination protocol in inducing the antibody immune response in BALF, once all piglets were challenged with the pathogen and an immune response was expected at 28 dpi. However, it is possible to observe that a substantial Ab detection occurred in piglets previously submitted to both IM and oral vaccination when compared to the control. It may be due not only to the capacity of vaccines to induce memory cells, but also to a previous local presence of these antibodies, as observed in the respiratory tract by nasal swabs. It was expected that vaccinated animals showed higher mucosal IgA Ab responses compared to the non-vaccinated ones after challenge^28,38^.

The number of organisms colonizing a pig possibly depends on cumulated infectious doses, capacity of the *M. hyopneumoniae* strain to multiply in the lungs, and time^1^. In the present study, a high dose of inoculum containing *M. hyopneumoniae* organisms was provided. *M. hyopneumoniae* replication in the lungs was lower in vaccinated pigs in both slaughter points compared to control, and quantification in lungs and BALF was lower at 56 dpi in vaccinated groups than in control, despite non-significant differences in bacterial load estimate quantification. The lower lung lesion score at 56 dpi was expected for the immunized groups, as previously observed^39^. However, it also indicates that vaccination alone does not significantly reduce the bacterial load in the lower respiratory tract of pigs, and would not eliminate infection with this pathogen from pig herds^24,27^, which may justify nasal shedding not differing between groups along the time^37^. In addition, several factors such as the challenge dose, the time post-infection, the strain virulence and individual immune responses of pigs may influence the number of *M. hyopneumoniae* organisms and its nasal shedding^23,33^.

The evaluation of cytokines production 30 days after challenge with *M. hyopneumoniae* could better illustrate the resistance status in the vaccinated pigs after infection^40^. Thus, in the current study, the expression of some cytokine genes at 28 dpi of vaccinated animals point out to a possible role of T cell response after pathogen exposure, so that the oral administration of *M. hyopneumoniae* antigens incorporated to the silica adjuvant may induce an activation of Treg lymphocytes, which can reduce part of the inflammatory response caused by this pathogen. In this context, it is known that mesenteric Treg lymphocytes of animals submitted to tolerance by the oral route secrete TGF-β with variable amounts of IL-4^41^. Additionally, TGF-β expression was positively correlated with IgA Ab response in the upper respiratory tract, raising the hypothesis that the presence of TGF-β further enhances both secreted IgA and the number of IgA producing cells, as reported^42^.

T helper (Th) cells are essential to initiate the B-cell activation and generation of antibody responses, which will result in antibody production for T-dependent antigens^5^. The higher gene expression level of IL-4 in lungs of immunized piglets could also be associated with a positive regulation of a Th2-mediated immune response^30^, positively influencing the IgA Ab local secretion. Th lymphocytes activation is an important pathway for generating protective immune response against *M. hyopneumoniae*^43^.

Interferon-γ secretion is important for the control of several infectious diseases^44^, and it is usually evident between 4 to 8 weeks post vaccination against *M. hyopneumoniae*^30,38^. Although the piglets were tested only at 28 days post-infection, its lower expression in immunized groups may be involved with an immunosuppressed environment caused by the inhibition of Th1 differentiation, since IL-4 suppresses the production of IFN-γ by Th1 cells^45^. In addition, IFN-γ was negatively correlated with IgA Ab response in the upper respiratory tract. These findings may be justified either by the suppressive effect of IL-4 or the effect of TGF-β on IFN-γ secretion and the direct correlation of IL-4 and IgA Ab response. IL-8 was found to be less expressed in all immunized groups of this study, and its suppression at this time point may be similar to IFN-γ. This chemokine is mainly produced by tissue macrophages in response to infection, and it is an important neutrophil recruiter^46^. *M. hyopneumoniae* organisms stimulate the production of proinflammatory and immunoregulatory cytokines by the alveolar macrophages and lymphocytes, inducing lung inflammation and lymphoid hyperplasia. As colonization by *M. hyopneumoniae* progresses, there is an increase in cytokine secretion due to the increase in the number of inflammatory cells recruited^1^. In non-vaccinated challenged piglets^23^, IL-8 gene expression was positively correlated with *M. hyopneumoniae* bacterial loads, suggesting an intense inflammatory response being required at lung lesion sites due to the presence of the pathogen. In our study, IL-8 and IFN-γ were found downregulated in vaccinated piglets, which may be due to modulation effect caused by the vaccines, preventing an intense inflammatory and immune responses in lungs, consequently leading to a lower consolidation lung lesion.

Under the conditions of this study, the immune response induced by IM and oral vaccination involved both humoral and likely T-cell immune responses. Thus, oral vaccination with a blend of *M. hyopneumoniae* antigens incorporated into the silica induced local humoral immunity in the gut, measured in this study as IgA anti-*M. hyopneumoniae* antibodies in respiratory secretions, which were comparable to those obtained by the intramuscular administration of the commercial inactivated vaccine with oil adjuvant. All vaccination protocols reduced the severity of macroscopic lung lesions in the challenged pigs. It suggests that mucosal antibodies and the inflammatory responses were involved in the mechanism of immune-protection, or by raising IL-4 and reducing the IL-8 expression, or even likely by down-regulating the expression of other pro-inflammatory cytokines in the vaccinated animals, which were not evaluated in the current study.

As the protection induced by *M. hyopneumoniae* vaccines evaluated here and conferred by others conventional vaccines do not prevent colonization and shedding of this microorganism, the immune-protection induced by these vaccines is often incomplete^47^. Considering that, better results may be achieved whether vaccination is combined with good management and biosecurity procedures in pig farms. Moreover, under field conditions, improved results should be expected for the vaccines tested in this study, once a lower infection challenge dose probably results in lower number of microorganisms in the respiratory tract^21^. Thus, future studies applying the oral vaccination against *M. hyopneumoniae* in pigs reared in field conditions could elucidate this point and bring more consistent results for the use of inactivated oral vaccines in the control of this important respiratory pathogen for pigs, combined with the advantage of being a needle-free strategy of *M. hyopneumoniae* control.

The oral vaccine developed in this research with the SBA-15 nanostructured silica proved to effectively reduce macroscopic lung lesions in challenged pigs and induce mucosal humoral immune response. Moreover, its efficacy was similar to the one conferred by the commercial vaccine parentally administered. The immunogenicity characterization determined in the current study provided useful data for the further development of this oral vaccine, which requires studies under field conditions to elucidate its potential for the effective control and prevention of *Mycoplasma hyopneumoniae* injuries in the pig production.

## Methods

### Oral vaccine preparation

#### Cultivation and preparation of *M. hyopneumoniae* for protein obtainment

A pure pathogenic strain of *M. hyopneumoniae* (232) was purchased from Iowa State University^48^, certified free of any other pathogens, and a small fraction was removed for cultivation in Friis medium. Initially, this fraction was inoculated into two sterile graduated vials (Corning®, USA) containing 5 ml of Friis medium, kept in a shaking oven at 37 °C, which color was observed daily until indicating bacterial growth (CCU). After approximately five days, 2 ml of the culture were used to inoculate each of two flasks containing 200 ml of Friis medium, which were maintained in the same incubation conditions and presented color changing about a week later. The determination of the concentration of *M. hyopneumoniae* was carried out by successive dilutions of the samples in Friis medium, varying from 10^–1^ to 10^–8^, which reached a concentration of 10^7^, and the negative control remained unchanged. Sterility tests were performed for all flasks on blood agar and McConkey media, left in an oven at 37 °C for three days, proving the absence of contamination with other pathogens by the absence of colonies growth.

The contents were centrifuged in appropriate tubes, previously autoclaved, in an ultracentrifuge (Sorvall) at 13,700×*g* for 45 min. The bacterial cells were deposited at the bottom of the tubes, forming pellets, which were resuspended in 15 mL of Phosphate Buffered Saline 1X (PBS, Sigma-Aldrich, USA), pH 7.4, for washing. The tubes were centrifuged three times at 21,000×*g* for 10 min until a clean pellet was obtained, which was resuspended in 10 mL of 1X PBS and stored in sterile falcon tubes. The resuspended content underwent a sonication process, and before sonication, an aliquot of whole cell preparation was taken for Dynamic Light Scattering (DLS) evaluation, which will be further discussed. For sonication, the flask was kept on ice and sonicated for three consecutive times in a sonicator (Soni-tech Ultrasonic Cleaning) in 20 Hz frequency for 1 min, with one-minute breaks between processes. To determine the concentration of proteins in the cell lysate, the Bradford method was used (Thermofisher Scientific, USA) followed by spectrophotometer reading (NanoDrop One, Thermofisher Scientific, USA), which provided a concentration of 1048 µg/mL.

#### Characterization and development of the oral vaccine

**Figure 6:**
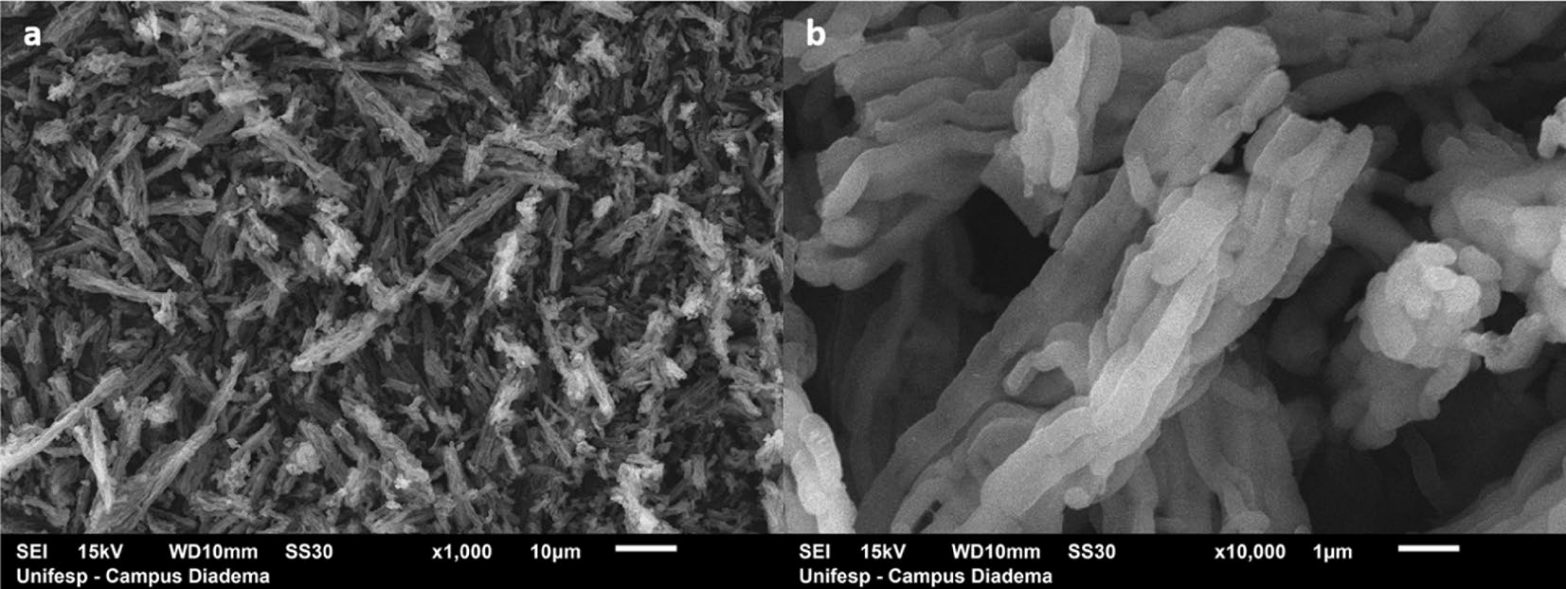
Scanning electron microscopy images of SBA-15 (Santa Barbara Amorphous silica) before antigen adsorption in macro pores. (**a**) 1000x magnification; (**b**) 10,000x magnification.

A partnership was established with the Department of Applied Physics, of the Physics Institute of the University of São Paulo (USP-SP), Department of Chemistry, Federal University of São Paulo (UNIFESP/Diadema) and the Butantan Institute (São Paulo), which have been conducting researches with an innovative immunogenic complex for human oral vaccines. They are based on the use of ordered mesoporous silica (OMS, SBA-15 type) as protective vehicle of antigens. The SBA-15 sample was synthesized according to Cavalcante^49^. It is composed by a bi-dimensional matrix of hexagonally ordered mesopores (diameter around 10 nm), high specific surface (around 1240 m^2^ g^−1^) and pore volume 1.8 cm^3^ g^−1^ (see Supplementary Fig. S1 online), with amorphous silica walls and rod-like morphology, formed by aggregates of rods connected as rope-like domains (Fig. 6), capable of encapsulating different molecules into their macropores and mesopores. The immunogenic complex has to be able to cross the digestive tract in order to be absorbed by the intestinal mucosa.

To protect the oral vaccine from the harsh stomach medium, the commercial polymer Eudragit® (Evonik Industries) was used for coating. This polymer is insoluble in acidic pH and dissolves in contact with the intestinal basic medium, providing slow release of the desired proteins^12^.

The preparation and characterization of the vaccine particles were carried out at the Crystallography Laboratory of the Department of Applied Physics at USP. The process of including the antigen in the pores of the SBA-15 was carried out considering the concentration of the obtained proteins. The 1:35 antigen-to-silica ratio was established (w/w), based on previous work with Hepatitis B encapsulation in SBA-15^18,20^. In the present study, the silica SBA-15, from a closed recipient (to avoid contamination and humidity) was weighed on a precision scale (± 1 μg), and immediately macerated with a glass stick. After that, the total amount of the silica was mixed with the flask containing the liquid antigen (concentration determined previously). The wet mixture was spread in a glass recipient and partially covered by a glass plate, and placed in an oven at 35 (± 2) °C for total drying. This drying procedure took 48 h. Then, the product was covered with Eudragit L30 D-55 (Evonik®), by mixing the polymer with the silica + antigen, using a glass spatula to homogenize the final product. The amount of Eudragit was equal (in weight) to the amount of silica added to the liquid antigen until a paste was formed, which was taken again to an oven at 35 (± 2) °C for drying, during 48 h. The dry contents were stored in a refrigerator to protect the antigen until use. Since the polymer has the ability to dissolve at a pH higher than 5.5, the compound was supplied to piglets in acidified water (pH 4.5).

#### Dynamic light scattering (DLS) evaluation of particle diameter and nitrogen adsorption/desorption isotherms of SBA-15:Antigen sample

Dynamic Light Scattering (DLS) evaluation was performed at the Physics Institute, USP-SP, using a DLS (DynaPro NanoStar, Wyatt) equipment at 90° scattering angle with a 100 mW He–Ne laser and wavelength of 658 nm. An aliquot of the whole-cells (concentration of 10^6^ CCU/mL) dispersed in 25 mL of PBS (pH 7.4) was used to fill the sample holder. The same process was done to investigate by DLS the sonicated sample (cell lysate), having a concentration of 1048 µg/ml. The size distribution was obtained by the equipment software, calculated by the CONTIN method^50^. The antigen encapsulation, depending on its size, can occur in the silica mesopores, with an average diameter of ~ 10 nm, or in the morphological macrospores, with dimensions greater than 50 nm. The average diameter of the molecules present in the whole-cell preparation sample was 460 nm, while the protein preparation was 218 nm, which allowed inferring that cell lysis occurred, and the antigen would be adsorbed on the silica macro porosity, regarding the sizes of the antigen in solution compared with the mean diameter of the mesopores.

By nitrogen adsorption/desorption isotherms of SBA-15:Antigen sample (Supplementary Figs. S2 and S3 online) were evidenced the preservation of mesostructured SBA-15, which exhibited a type IV isotherm and type H1 hysteresis loops, according to the IUPAC classification^51^ that are characteristic of hexagonal cylindrical channel mesoporous such as SBA-15, with a pore size about 10 nm, as reported in the literature^52^. The reduction of specific surface area (336 m^2^ g^−1^) and pore volume (0.91 cm^3^ g^−1^) of SBA-15:Antigen, comparing to pure SBA-15, has evidenced antigen into the macropores of SBA-15.

#### Oral vaccine administration

After a pilot test, a concentration of 200 µg of protein antigen of *M. hyopneumoniae* per animal was established. The doses of the compound were weighed on a precision scale, considering the proportion between antigen, silica and polymer. The vaccine was provided by gavage using an esophageal tube with the aid of a laryngoscope, administered in individual doses diluted in 5 ml of filtered water plus acidifier (Selko pH, Trouw Nutrition). Each dose was diluted at the moment of administration.

### Experimental design and sample collection

#### Experimental design

Fifty piglets were randomly separated into five groups (n = 10) to receive different vaccine protocols, consisting of a commercial vaccine (CV) or oral immunization (OI). Random numbers were generated using the standard = *RAND*() function in Microsoft Excel*.* Group 1 (CV) piglets received a single dose commercial vaccine at 24 days of age (D0). Group 2 (OI) piglets received a single dose of oral vaccine at D0. Group 3 (CV + OI) received a dose of the commercial vaccine at D0 and a booster with the oral vaccine at D28. Group 4 (OI + OI) received a dose of the oral vaccine on D0 and a booster with the same vaccine on D28; piglets of Group 5 (CONT) were the control group, which did not receive any form of immunization. The commercial vaccine chosen for this work was M+PAC (Merck Animal Health, USA), which promotes an effective protection against *M. hyopneumoniae* as it contains aluminum hydroxide, responsible for a rapid immune response, and an oil emulsion, which promotes a prolonged immune response by its slow release in the organism. At about 70 days of age (D49), all piglets were challenged by the tracheal route with 5 mL of Friis medium containing 10^6^ CCU/mL of *M. hyopneumoniae* virulent strain 232, homologous to the vaccine.

Weekly, nasal swabs were collected since D0 for IgA measurement (ELISA), and, after the challenge, were used to evaluate *M. hyopneumoniae* shedding as well. Fortnightly, all piglets underwent blood collections to obtain serum for IgG measurement (ELISA). Half of the animals in each group were euthanized 28 days post-infection (D77), and the other half was euthanized 56 days post-infection (D105). At slaughter, lungs of all animals were macroscopically evaluated following the European Pharmacopeia methodology, biological samples such as lung fragments were collected for qPCR and histopathology, and bronchoalveolar fluid (BALF) for qPCR. A schematic design is shown in the Supplementary Fig. S4 online.

#### Animal selection

Fifty 21-day-old piglets of commercial lineage (Landrace × Large White) and average weight between 6 and 7 kg were purchased from a certified *M. hyopneumoniae*-free commercial farm. The piglets were housed in the experimental barn of the Swine Medicine Laboratory of School of Agricultural and Veterinarian Sciences (FCAV), remaining in the nursery pens until 65 days of age (3 pigs/m^2^), and then transferred to fattening pens (1 pig/m^2^), where they remained until 130 days of age. Biosecurity standards were met to avoid cross-infections and minimize external interferences, such as shower-in/shower-out, specific and clean clothes for pig husbandry and no contact with any other pig during the experimental period. The animals received feed according to the production phase, free of antibiotics, and water ad libitum. Upon arrival, blood samples were taken from all piglets to obtain serum for measurement of antibodies, in addition to laryngeal swabs to confirm the absence of the pathogen. Piglets went through an adaptation period of three days before the beginning of the experiment.

All procedures described here were conducted in accordance with the Federal Council of Veterinary Medicine (Brazil), submitted for approval by the Ethics Committee on the Use of Animals (CEUA) of the School of Agricultural and Veterinarian Sciences, São Paulo State University—Campus Jaboticabal, being approved and registered under the protocol number 005174/18. The study was carried out in compliance with the ARRIVE guidelines^53^.

#### Blood serum and nasal swabs collection

The titers and duration of antibodies in serum and nasal swabs were assessed along the experimental period. The blood was collected every two weeks to obtain blood serum by puncture the jugular vein, using sterile disposable needles and syringes, deposited in tubes with clot activator and centrifuged at 1500×*g* for 10 min. The serum was aliquoted in duplicate and stored at − 20 °C until the time of analysis. Samples of nasal swabs were weekly collected to obtain quantitative data on the immune response induced by different protocols of immunization (ELISA), and on the dynamics of excretion of *M. hyopneumoniae* (qPCR). The animals were restrained, and the swab samples were collected from a light rubbing of the swab on the nasal mucosa and deposited in 2 mL graduated plastic microtubes (Kasvi, Brazil) containing 500 μL of 1 × PBS, and stored at − 80 °C until analysis.

#### Slaughtering of piglets, lung lesion scoring, BALF and lung fragments collection

Four and eight weeks after the challenge, half of the piglets of each group was euthanized with an intramuscular administration of a combination of ketamine and xylazine (6 mg/kg and 4 mg/kg, respectively), followed by intravenous administration of saturated potassium chloride solution (approved by Federal Council of Veterinary Medicine, Brazil). After evisceration, the respiratory set (trachea + lung) of each animal was separated to collect bronchoalveolar fluid (BALF), with the introduction of 20 mL of PBS 1 × in the cranial portion of the trachea. After pouring all the liquid, the lung was lightly massaged and the liquid was aspirated by the pipette, recovering an approximate volume of 10 mL. The aspirate was aliquoted in duplicate in 2 mL graduated microtubes, free of DNAses and RNAses (Kasvi, Brazil), while the remaining volume was deposited in sterile Falcon tubes (Kasvi, Brazil) and stored at – 20 °C until analysis by qPCR.

The lung was evaluated for the extent of lung lesions followed by photo documentation by a unique blinded investigator, and the extent of the lesions was quantified using the European Pharmacopeia method, in which the percentage of each lobe affected area is multiplied by the lobe relative weight and summed to provide the total weight percentage of affected lung^54^. The lung tissue fragments for the qPCR were collected with individual scalpel blades and sterile tweezers. The tweezers were kept in boiling water in the interval between collections, while each fragment was collected with a disposable scalpel blade. These fragments were collected in duplicate in the shortest possible time after the animal's death, and all samples were bathed in liquid nitrogen and later stored in a freezer at − 80 °C.

### Histopathological evaluation

The histopathological analysis aimed at evaluating lung lesions caused by *Mycoplasma hyopneumoniae* in different groups, in order to classify qualitatively histological lesions as PEP-specific or non-specific. For that, during the necropsy, lung fragments with lesions caused by PEP resources were collected, mainly in the transition area between healthy and affected tissue. Samples of tissues apparently healthy were also collected for control. The fragments were collected with a thickness of approximately 5 mm and initially stored submerged in a 10% buffered formalin solution (pH 7.0) in an approximate ratio of 10: 1 formalin: tissue. After 24 h in formalin solution, the fragments were routinely processed for Hematoxylin/Eosin staining.

The slides were blind read under a light microscope and microscopic lesions on the tissues were classified into five different degrees^55^, in which: 0 = absence of lesion; 1 = lesions of interstitial pneumonia and/or purulent bronchopneumonia; 2 = light to moderate infiltrates of macrophages, lymphocytes and neutrophils into airways and alveoli; 3 = perivascular and peribronchiolar lymphoplasmacytic hyperplasia, type II pneumocyte hyperplasia, alveolar spaces with edema fluid, neutrophils, macrophages and plasma cells; 4 = lesions with characteristics of grade 3, together with peribronchial and perivascular lymphoid nodules. Grades 1 and 2 injuries were nonspecific, while injuries 3 and 4 were considered PEP-specific.

### Detection and quantification of IgA and IgG antibodies by enzyme-linked immunosorbent assay

The detection and quantification of antibodies (IgA and IgG) in blood serum, nasal secretions and tracheobronchial lavage were carried out by enzyme-linked immunosorbent assay (ELISA). To detect serum IgG Ab, the commercial kit *M.hyo Ab test* (Idexx, USA) was used. For the detection of IgA Ab in nasal swab, and IgA and IgG Abs in BALF samples, standardization was performed using the sensitized plates and components of the kit mentioned above, with modifications. Initially, the plate was blocked with 1.5% ovalbumin in PBS, followed by incubation at 37 °C for 30 min. Then, a first procedure was performed to determine the ideal dilutions of the samples (positive and negative controls for anti-*M. hyopneumoniae* antibodies) and the anti-IgA or anti-IgG peroxidase conjugates. For standardization, samples of nasal swabs from animals known to be positive and negative for *M. hyopneumoniae* were used, and part of the negative and positive samples were homogenized in the form of a pool to compose the negative (CN) and positive (CP) controls, respectively.

To detect IgA Ab in nasal swabs, 100 μL of the sample's liquid fraction was used, as the swabs were deposited in 500 μL of PBS, which were quickly homogenized in a vortex and placed, without further dilution, in each microplate well. The same procedure was performed with the controls, which were tested in duplicate, followed by plate incubation for 60 min at room temperature. The conjugate from the kit was replaced by an immunoenzymatic conjugate of goat anti-Pig IgA Antibody HRP Conjugated (Bethyl Laboratories Inc., USA), at a dilution of 1:500 using the diluent provided by the kit, followed by incubation for 60 min at room temperature. The washing processes and all the following steps were performed according to the protocol of the kit *M.hyo Ab test* (Idexx, USA).

For BALF IgA Ab detection, the samples were diluted 1:10 using the diluent from the kit, and the same conjugate was used in the proportion of 1:800. For IgG detection in the BALF, the samples were diluted in the proportion of 1:2 and the conjugate (Pig IgG-Fc Fragment Antibody HRP, Bethyl Laboratories Inc., USA) in the proportion of 1:5000. In all microplates, the conjugate was tested in separate wells to determine its non-specific adsorption in the absence of samples. The plate was read in an absorbance microplate reader (iMark, Bio-Rad Laboratories Inc., USA), at a wavelength of 650 nm.

The mean optical densities (OD) for each of the test samples (*ODs*) were related with the OD found for the negative and positive controls (*NC*$\overline{x }; PC\overline{x })$ in order to calculate the S/P values (sample/positive ratio) according to the formula: S/P = $ODs- NC\overline{x }/PC\overline{x }- NC\overline{x }$. The threshold between positive and negative samples was calculated from the value of S/P $NC\overline{x }$ + 2 × standard deviation. Serum samples were considered positive if S/P > 0.3; nasal swabs S/P > 0.4. BALF S/P > 0.4.

### Detection and quantification of *p102* gene fragment of* M. hyopneumoniae* by qPCR

#### DNA extraction from nasal swabs, lung and BALF samples

The DNA extraction was carried out by Tris–HCl protocol^56^. For nasal swabs and BALF samples, a centrifugation (Centrifuge 5804 R, Eppendorf, Germany) at 13,000×*g* at 4 °C for 20 min was performed previously to the DNA extraction protocol. For lung samples, 0.05 g of lung tissue were used. After DNA extraction, the samples were stored at − 20 °C until qPCR analysis. The measurement of the DNA concentration of the samples was made through spectrophotometry, with the aid of the Thermo Scientific NanoDrop 2000 Spectrophotometer (Thermo FisherScientific®, USA), having as exclusion factor the samples that did not reach the purity of 1.8 to 2.0 in the 260/280 ratio to perform the qPCR technique. To rule out the presence of inhibitors in the extracted DNA samples and the occurrence of false negatives in the qPCR for *M. hyopneumoniae*, all samples were submitted to a conventional PCR targeting the endogenous gene Glyceraldehyde-3-phosphate dehydrogenase (*gapdh*), and the conventional PCR technique^57^. The amplified products of *gapdh* gene with 437 bp were detected after horizontal electrophoresis on a 1% agarose gel stained with Ethidium Bromide (0.5 μL/mL) in TEB running buffer pH 8.0 at a current of 90 V/50 mA for 90 min.

#### qPCR assay

Absolute real-time quantitative PCR analysis (qPCR) was used to detect *p102* gene fragment in nasal swab samples, and to detect and quantify it in lung fragments and bronchoalveolar fluids. For *M. hyopneumoniae*, the primers used in the reaction were based on the bacterium *p102* adhesion protein gene sequence. All samples were tested in duplicate and the qPCR reaction was optimized from a previous published protocol^58^, adapted by Almeida^23^. The nucleotide sequences used were forward primer 5′-AAGGGTCAAAGTCAAAGTC-3′, reverse primer 5′-AAATTAAAAGCTGTTCAAATGC-3′ and hydrolysis probe 5′-FAM-AACCAGTTTCCACTTCATCGCC-§BHQ2-3′.

The results were only accepted for those with a standard deviation lesser than or equal to 0.5 cycle, and quantification data were used only if the efficiency obtained was between 90 and 105%^57^, otherwise, the samples were retested in triplicates. As a negative control in the qPCR reactions, sterile ultrapure water was used (Nuclease-Free Water, Promega®, Madison, Wisconsin, USA) q.s.p. Serial dilutions were made to determine the standard curve generated with different concentrations of synthetic DNA (GBlock®, IDT, USA) containing the target sequence (10^7^ copies/μL to 10^1^ copies/μL), that were also used as positive controls. The synthetic DNA was diluted according to the manufacturer's guidelines and maintained in a stock concentration of 10^7^ molecules∕µL.

Quantification was performed using serial tenfold dilutions (starting at 10^7^ until 10^1^ copies∕ μL) of synthetic DNA (GBlock®, IDT, Iowa City, IA, USA) containing the 150 bp fragment amplified by the primer pair used in the qPCR. Quantification data based on the standard curve generated was only validated if the reaction efficiency was between 90 and 105%^59^. qPCR parameters are shown in Supplementary Table S1.

### Cytokine coding gene expression in lung samples

#### RNA extraction and cDNA synthesis

Total RNA was extracted from 0.02 g of lung tissue samples collected on the first slaughter (28 dpi) using RNeasy Blood and Tissue Plus kit (Qiagen, USA), and 500 ng of extracted RNA were used per reaction to convert the extracted RNA into cDNA by Superscript IV First Strand Synthesis kit (Thermo Fisher, USA), both according to the manufacturer’s guidelines. RNA purity and integrity were assessed by Bioanalyzer® (Thermo Scientific, USA), and immediately stored at − 80 °C until use. Samples were only used for gene expression analysis if they had RNA Integrity Number (RIN) > 7.0. Oligo d(T)20 targeted the poly-A tail of mRNA for the synthesis of this molecule into cDNA, instead of other types of RNA. Reactions were all performed in a MyCycler thermocycler (Bio Rad, USA), and the cDNA was stored at − 20 °C until use for qPCR.

#### Detection and quantification of cytokine coding gene’s expression

The reference gene used to normalize the expression of target genes was standardized^23^, and the best result was obtained with Ribosomal protein L4 (rpl-4), which was also used in this study. Quantification of cytokine coding gene’s expression was performed using relative quantification of cDNA produced. Transcript levels of inflammatory (IL-8) and specific immunity regulation (IFN-γ IL-4 and TGF-β) cytokines evaluated to compare the immune response promoted by each type of immunization with the control group. Specific primers targeting the genes of IL-8 (CXCL-8) and IFN-γ were based on previous work^23^, while genes of IL-4 and TGF- β were designed based on the reference sequences deposited in GenBank and using software Primer3^60^. To avoid genomic DNA interference, all primers were designed comprising the exon-exon span. The specific primers are shown in Table 3.

**Table 3:** Details of the primer sequences of cytokines used for quantitative SYBR Green real-time PCR amplification. The *rpl-4* gene was used as reference.

Target gene	Primer sequence	Access number	qPCR efficiency (%)	Slope	R^2^	Amplicon size (bp)	Amplicon melting temperature (°C)
*rpl-4*	(F)CAAGAGTAACTACAACCTTC	NC_010443.5	96.79	3.401	0.994	122	75
	(R)GAACTCTACGATGAATCTTC						
IL-8	(F)AGGAAAAGTGGGTGCAGAAG	NM_213867.1	97.67	3.379	0.991	190	78
	(R)CAACCCTATGTCTGACCAGC						
IFN-γ	(F)ATTGGAAAGAGGAGAGTGAC	NM_213948.1	96.54	3.408	0.997	168	76.5
	(R)CATTCAGTTTCCCAGAGCTA						
IL-4	(F)AGAGCTCTATTCATGGGTCT	NM_214123.1	99.88	3.325	0.997	209	82
	(R)CTTCTCCGTCGTGTCTCT						
TGF-β	(F)GGATACCAACTACTGCTTCA	NM_214015.2	100.85	3.302	0.996	228	83
	(R)TTGTACAGAGCCAGGACTT						

The qPCR reactions were performed as previously described^23^, using the Quantitect® Sybr Green master mix (Qiagen, USA) and 1 μL of cDNA template, totalizing 10 μL of final volume per reaction, in a real time thermocycler CFX 96 (Bio Rad, USA). The dissociation curve was used to assess the specificity of the amplicons at the end of 40 cycles, with a maximum variation of ± 0.5 °C. Serial tenfold dilutions (10^7^ copies∕μL until 10^1^ copies∕μL) of positive controls (Gblock®, IDT, USA) of synthetic DNA containing the amplified fragment of each primer pair was used to assess the efficiency, which was only accepted between 90 and 105%^59^. In order to normalize the target gene expression, the 2^−ΔΔCq^ calculation^61^ was performed, and, to attend this methodology, all qPCR reaction’s efficiency had to be close to 100% with a maximum difference of 5% between them. All parameters of cytokine gene expression qPCR are shown Supplementary Table S2.

### Data analysis

The variables of each group for each moment were assessed for normality and homoscedasticity by the Shapiro–Wilk and Bartlet tests, respectively. The difference between the means was calculated using the Tukey test (p < 0.05). Variables that did not meet the assumptions were subjected to the Kruskal–Wallis non-parametric test (p < 0.05) and in cases which significance was observed, the Dunn test (Post hoc) was applied. Differences between the slaughter times for the quantitative variables were subjected to parametric analysis by the unpaired T-test, and the variables that did not meet the assumptions were subjected to the Wilcoxon–Mann–Whitney non-parametric test. The difference between the means (post hoc) was calculated by the last square mean adjusted by the Tukey method. For proportion analysis, the differences between groups by date were calculated using the Wilson score interval method. The correlation analysis of parametric data were subjected to Pearson's correlation test (p < 0.05), and non-parametric data, to Spearman's test. Correlation analysis to assess whether two variables can be considered dependent was performed using Kendall's nonparametric test. For this, the software R was used, with the following packages “agricolae”^62^; "LmerTest"^63^, "arm"^64^; "Emmeans"^65^; "Car"^66^; "Nortest"^67^; "MASS"^68^ with the software R^69^. Normalization of cytokine gene expression and the graphs were performed on software GraphPad Prism 6 (La Jolla, CA-USA).

## Supplementary Information

Supplementary Information.

## Data Availability

The datasets generated during and/or analysed during the current study are available from the corresponding author on reasonable request.
