# Supplementary material for: Oral vaccination of piglets against Mycoplasma hyopneumoniae using silica SBA-15 as an adjuvant effectively reduced consolidation lung lesions at slaughter
Source: Sci Rep. 2021 Nov 17;11:22377. doi: 10.1038/s41598-021-01883-2 (PMC8599662; doi:10.1038/s41598-021-01883-2)
Supplement: Supplementary file 1 — Supplementary Information. [file 41598_2021_1883_MOESM1_ESM.docx]

**Supplementary material**

**Oral vaccination of piglets against** *Mycoplasma hyopneumoniae* **using silica SBA-15 as an adjuvant effectively reduced consolidation lung lesions at slaughter**

**Marina L. Mechler-Dreibi^1*^; Henrique M. S. Almeida^1^; Karina Sonalio^1^; Mariela A. C. Martines^1^; Fernando A. M. Petri^1^; Beatriz B. Zambotti^1^; Marcela M. Ferreira^1^; Gabriel Y. Storino^1^, Tereza S. Martins^2^; Hélio J. Montassier^1^; Osvaldo A. Sant’Anna^3^; Márcia C. A. Fantini^4^; Luís Guilherme de Oliveira^1*^**

^1^São Paulo State University (Unesp), School of Agricultural and Veterinarian Sciences, Jaboticabal, Brazil.

^2^Department of Chemistry, Federal University of São Paulo (UNIFESP), Diadema, SP, Brazil.

^3^Butantan Institute, São Paulo, Brazil.

^4^University of São Paulo (USP), Physics Institute, São Paulo, Brazil.

*** Correspondence:**Corresponding Authors: [mlopesvet@gmail.com](mailto:mlopesvet@gmail.com), [luis.guilherme@unesp.br](mailto:luis.guilherme@unesp.br)

**Small-angle X-ray scattering (SAXS)**

**Supplementary Figure S1.** SAXS Curves of SBA-15. *SAXS measurements were performed using a Nanostar (Bruker) instrument equipped with a microfocus Genix 3D system (Xenocs). The powder samples were accommodated in a sample holder between two mica slices, and the scattered intensity was collected on a 2D Vantec-2000 detector***.**

**Adsorption-desorption isotherms (NAI)**

**Supplementary Figure S2.** Adsorption-desorption isotherms in N_2_ at 77K (A) and pore size distribution (B) of SBA-15 sample. *NAI measurements were performed with a Quantachrome (NOVA 1200e) porosimeter at 77 K using nitrogen of 99.998% purity.*

**Supplementary Figure S3.** Adsorption-desorption isotherms in N_2_ at 77K (A) and pore size distribution (B) of SBA-15:Antigen sample. *NAI measurements were performed with a Micromeritics ASAP 2020 porosimeter at 77 K using nitrogen of 99.998% purity.*

***
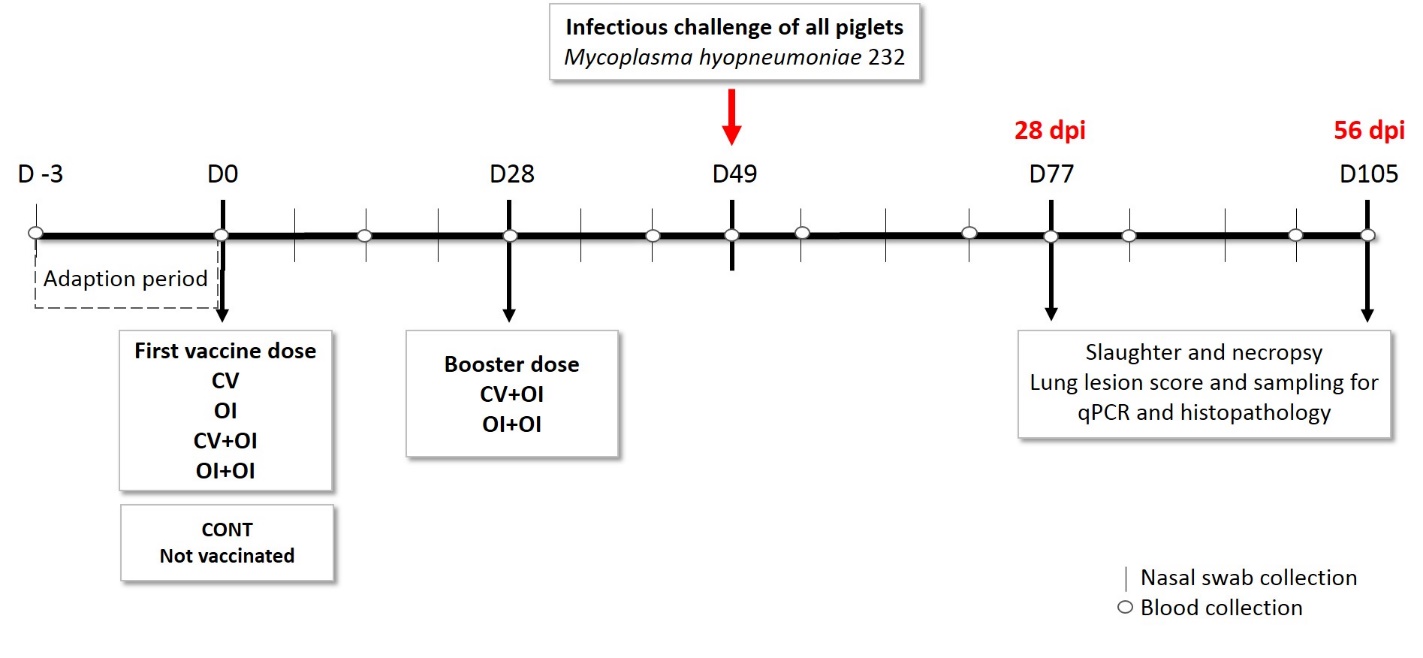
***

**Supplementary Figure S4.** Experimental design with point by point of the data collection, slaughter of animals and collection of biological samples of piglets challenged with *Mycolpasma hyopneumoniae* 232. CV= Single dose of commercial vaccine; OI= Single dose of oral immunization; CV+OI= One dose of commercial vaccine and a booster with the oral immunization; OI+OI= One dose and a booster with the oral immunization; CONT= control group, not vaccinated.

**Supplementary Table S1.** Detailed parameters of each qPCR assay for lung estimate *Mycoplasma hyopneumoniae* quantification.

| **Plate no.** | **Efficiency** | **Slope** | **R^2^** | **y-intercept** |
| --- | --- | --- | --- | --- |
| **1** | 97.6% | 3.381 | 0.995 | 39.34 |
| **2** | 102% | 3.275 | 0.999 | 37.98 |
| **3** | 100.1% | 3.320 | 0.998 | 38.08 |
| **4** | 100% | 3.322 | 0.999 | 36.89 |
| **5** | 102.2% | 3.269 | 0.997 | 38.57 |
| **6** | 98.8% | 3.351 | 0.999 | 37.77 |
| **7** | 103.1% | 3.250 | 0.998 | 39.33 |
| **8** | 101.9% | 3.278 | 0.997 | 38.88 |
| **9** | 100.4% | 3.313 | 0.998 | 38.45 |
| **10** | 103.8% | 3.233 | 0.998 | 38.23 |

**Supplementary Table S2.** Detailed parameters of each qPCR assay for cytokine gene expression in lung fragments of piglets challenged with *Mycoplasma hyopneumoniae*.

| **Plate no.** | **Efficiency** | **Slope** | **R2** | **y-intercept** |
| --- | --- | --- | --- | --- |
| **1. IL-4** | 99.88% | 3.225 | 0.997 | 35.35 |
| **2. IL-8** | 97.67% | 3.379 | 0.991 | 39.98 |
| **3. IFN-γ** | 96.54% | 3.408 | 0.997 | 40.41 |
| **4. RPL-4** | 96.79% | 3.401 | 0.994 | 36.85 |
| **5. TGF-β** | 100.85% | 3.302 | 0.996 | 35.39 |

**Supplementary Table S3**. Mean values of IgA antibody quantification by ELISA in nasal swabs of piglets submitted to different immunization protocols, with the respective standard deviation values for each group and day of sampling. After D(77), each group remained with 50% of the piglets. Means followed by the same letter did not differ statistically in Tukey test.

|  |  | **CV** | **OI** | **CV+OI** | **OI+OI** | **CONT** |
| --- | --- | --- | --- | --- | --- | --- |
| **D0** | Mean | 0.185 ^a^ | 0.202 ^a^ | 0.189 ^a^ | 0.142 ^a^ | 0.154 ^a^ |
|  | SD | 0.140 | 0.184 | 0.187 | 0.092 | 0.129 |
| **D7** | Mean | 0.324 ^a^ | 0.270 ^a^ | 0.349 ^a^ | 0.484 ^a^ | 0.231 ^a^ |
|  | SD | 0.165 | 0.119 | 0.383 | 0.277 | 0.114 |
| **D14** | Mean | 0.565 ^a^ | 0.512 ^a^ | 0.547 ^a^ | 0.625 ^a^ | 0.308 ^a^ |
|  | SD | 0.163 | 0.247 | 0.316 | 0.457 | 0.134 |
| **D21** | Mean | 0.490 ^a^ | 0.345 ^ab^ | 0.369 ^ab^ | 0.349 ^ab^ | 0.212 ^b^ |
|  | SD | 0.301 | 0.117 | 0.200 | 0.198 | 0.096 |
| **D28** | Mean | 0.406 ^b^ | 0.316 ^b^ | 0.438 ^b^ | 0.772 ^a^ | 0.321 ^b^ |
|  | SD | 0.183 | 0.109 | 0.271 | 0.435 | 0.134 |
| **D35** | Mean | 0.555 ^a^ | 0.476 ^a^ | 0.572 ^a^ | 0.567 ^a^ | 0.328 ^a^ |
|  | SD | 0.227 | 0.214 | 0.303 | 0.339 | 0.138 |
| **D42** | Mean | 0.680 ^a^ | 0.480 ^ab^ | 0.464 ^bc^ | 0.393 ^bc^ | 0.274 ^c^ |
|  | SD | 0.189 | 0.145 | 0.170 | 0.255 | 0.082 |
| **D49** | Mean | 0.655 ^a^ | 0.419 ^bc^ | 0.590 ^ab^ | 0.379 ^bc^ | 0.213 ^c^ |
|  | SD | 0.173 | 0.223 | 0.247 | 0.174 | 0.089 |
| **D56** | Mean | 0.864 ^a^ | 0.514 ^b^ | 0.518 ^b^ | 0.435 ^bc^ | 0.183 ^c^ |
|  | SD | 0.272 | 0.203 | 0.325 | 0.270 | 0.126 |
| **D63** | Mean | 1.002 ^a^ | 0.800 ^ab^ | 0.806 ^ab^ | 0.561 ^bc^ | 0.300 ^c^ |
|  | SD | 0.172 | 0.344 | 0.298 | 0.306 | 0.203 |
| **D70** | Mean | 1.114 ^a^ | 0.684 ^b^ | 1.030 ^a^ | 0.522 ^bc^ | 0.220 ^c^ |
|  | SD | 0.262 | 0.319 | 0.251 | 0.380 | 0.189 |
| **D77** | Mean | 1.087 ^a^ | 0.548 ^ab^ | 0.946 ^ab^ | 0.458 ^b^ | 0.471 ^b^ |
|  | SD | 0.159 | 0.230 | 0.267 | 0.471 | 0.190 |
| **D84** | Mean | 1.152 ^a^ | 0.689 ^bc^ | 1.036 ^ab^ | 0.564 ^c^ | 0.397 ^c^ |
|  | SD | 0.122 | 0.229 | 0.232 | 0.358 | 0.174 |
| **D91** | Mean | 1.086 ^a^ | 0.752 ^a^ | 0.925 ^a^ | 0.937 ^a^ | 0.763 ^a^ |
|  | SD | 0.159 | 0.251 | 0.430 | 0.360 | 0.225 |
| **D98** | Mean | 1.465 ^a^ | 0.948 ^a^ | 1.370 ^a^ | 0.870 ^a^ | 0.902 ^a^ |
|  | SD | 0.257 | 0.611 | 0.216 | 0.333 | 0.211 |
| **D105** | Mean | 1.500 ^a^ | 1.199 ^a^ | 1.400 ^a^ | 1.174 ^a^ | 1.111 ^a^ |
|  | SD | 0.207 | 0.421 | 0.279 | 0.331 | 0.203 |

**Supplementary Table S4**. Mean values of IgG antibody quantification by ELISA in serum samples of piglets submitted to different immunization protocols, with the respective standard deviation values for each group and day of sampling. After D(77), each group remained with 50% of the piglets. Means followed by the same letter did not differ statistically in Tukey test.

|  |  | **CV** | **OI** | **CV+OI** | **OI+OI** | **CONT** |
| --- | --- | --- | --- | --- | --- | --- |
| **D0** | Mean | -0.096 ^a^ | -0.096 ^a^ | -0.096 ^a^ | -0.098 ^a^ | -0.100 ^a^ |
|  | SD | 0.023 | 0.026 | 0.027 | 0.018 | 0.021 |
| **D14** | Mean | -0.024^a^ | -0.070 ^a^ | 0.028 ^a^ | -0.236 ^b^ | -0.232 ^b^ |
|  | SD | 0.060 | 0.056 | 0.157 | 0.041 | 0.058 |
| **D28** | Mean | 0.393 ^a^ | -0.235 ^b^ | 0.372 ^a^ | -0.223 ^b^ | -0.227 ^b^ |
|  | SD | 0.456 | 0.043 | 0.392 | 0.048 | 0.049 |
| **D42** | Mean | 0.369 ^a^ | -0.088 ^b^ | 0.606 ^a^ | -0.108 ^b^ | -0.118 ^b^ |
|  | SD | 0.309 | 0.030 | 0.333 | 0.030 | 0.038 |
| **D49** | Mean | 0.426 ^a^ | -0.027 ^b^ | 0.472 ^a^ | 0.019 ^b^ | -0.110 ^b^ |
|  | SD | 0.362 | 0.215 | 0.410 | 0.301 | 0.020 |
| **D56** | Mean | 0.532 ^a^ | -0.079 ^b^ | 0.665 ^a^ | -0.083 ^b^ | -0.092 ^b^ |
|  | SD | 0.295 | 0.052 | 0.346 | 0.036 | 0.060 |
| **D70** | Mean | 1.273 ^a^ | 0.410 ^b^ | 1.568 ^a^ | 0.225 ^b^ | 0.229 ^b^ |
|  | SD | 0.499 | 0.242 | 0.510 | 0.306 | 0.404 |
| **D77** | Mean | 1.616 ^a^ | 0.619 ^b^ | 1.606 ^a^ | 0.263 ^b^ | 0.334 ^b^ |
|  | SD | 0.382 | 0.252 | 0.543 | 0.368 | 0.396 |
| **D84** | Mean | 2.034 ^a^ | 0.721 ^b^ | 1.633 ^a^ | 0.385 ^b^ | 0.505 ^b^ |
|  | SD | 0.408 | 0.242 | 0.568 | 0.479 | 0.432 |
| **D98** | Mean | 2.147 ^a^ | 0.999 ^b^ | 1.860 ^a^ | 0.743 ^b^ | 0.708 ^b^ |
|  | SD | 0.102 | 0.110 | 0.222 | 0.398 | 0.299 |
| **D105** | Mean | 2.203 ^a^ | 1.151 ^b^ | 1.903 ^a^ | 0.819 ^b^ | 0.753 ^b^ |
|  | SD | 0.110 | 0.105 | 0.218 | 0.268 | 0.270 |

**Supplementary Table S5**. Significant p-values on Tukey test for the comparison of nasal IgA results between groups of piglets submitted to different immunization protocols. Only time points with significant results are shown.

|  | **D21** | **D28** | **D42** | **D49** | **D56** | **D63** | | **D70** | | **D77** | | **D84** | |
| --- | --- | --- | --- | --- | --- | --- | --- | --- | --- | --- | --- | --- | --- |
| **OI/CV** | **-** | - | - | 3.43x10^-2^ | 1.15x10^-2^ | | - | | 7.99x10^-3^ | | - | 4.12.10^-2^ |  |
| **CV+OI/CV** | **-** | - | 3.82x10^-2^ | - | 1.27x10^-2^ | | - | | - | | - | - |  |
| **OI+OI/CV** | **-** | 1.01x10^-2^ | 2.52x10^-3^ | 8.50x10^-3^ | 1.11x10^-3^ | | 2.69x10^-3^ | | 8.17x10^-5^ | | 1.29x10^-2^ | 4.21.10^-3^ |  |
| **CONT/CV** | 9.91x10^-3^ | - | 1.04x10^-5^ | 7.91x10^-6^ | - | | 9.86x10^-7^ | | 8.59x10^-9^ | | 1.52x10^-2^ | 2.62.10^-4^ |  |
| **CV+OI/OI** | **-** | - | - | - | - | | - | | 4.35x10^-2^ | | - | - |  |
| **OI+OI/OI** | **-** | 5.51x10^-4^ | - | - | - | | - | | 6.64x10^-1^ | | - | - |  |
| **CONT/OI** | **-** | - | 4.61x10^-2^ | - | 1.54x10^-2^ | | 5.05x10^-4^ | | 2.72x10^-3^ | | - | - |  |
| **OI+OI/CV+OI** | **-** | 1.93x10^-2^ | - | - | - | | - | | 6.32x10^-4^ | | - | 1.89.10^-2^ |  |
| **CONT/CV+OI** | **-** | - | - | 9.62x10^-5^ | 1.39x10^-2^ | | 3.04x10^-4^ | | 6.54x10^-8^ | | - | 1.09.10^-3^ |  |
| **CONT/OI+OI** | - | 6.48x10^-4^ | - | - | - | | - | | - | | - | - |  |

**Supplementary Table S6**. Significant p-values on Tukey test for the comparison of serum IgG results between groups of piglets submitted to different immunization protocols. Only time points with significant results are shown.

|  | **D14** | **D28** | **D42** | **D49** | **D56** | **D70** | **D77** | **D84** | **D98** | **D105** |
| --- | --- | --- | --- | --- | --- | --- | --- | --- | --- | --- |
| **OI/CV** | - | 6.22x10^-6^ | 1.45x10^-5^ | 6.90x10^-3^ | 2.37x10^-8^ | 1.17x10^-4^ | 4.36x10^-3^ | 9.66x10^-4^ | 1.91x10^-5^ | 1.93x10^-5^ |
| **CV+OI/CV** | - | - | - | - | - | - | - | - | - | - |
| **OI+OI/CV** | 2.43x10^-6^ | 9.21x10^-6^ | 6.09x10^-6^ | 1.58x10^-2^ | 1.98x10^-8^ | 1.17x10^-6^ | 5.88x10^-5^ | 2.95x10^-5^ | 1.77x10^-7^ | 1.48x10^-7^ |
| **CONT/CV** | 3.63x10^-6^ | 8.04x10^-6^ | 4.03x10^-6^ | 9.25x10^-4^ | 1.34x10^-8^ | 2.01x10^-6^ | 1.27x10^-4^ | 8.43x10^-5^ | 2.42x10^-7^ | 2.23x10^-7^ |
| **CV+OI/OI** | - | 7.75x10^-6^ | 2.28x10^-10^ | 1.75x10^-3^ | 3.63x10^-11^ | 3.46x10^-7^ | 2.60x10^-3^ | 2.00x10^-2^ | 4.81x10^-4^ | 2.03x10^-4^ |
| **OI+OI/OI** | 1.55x10^-4^ | - | - | - | - | - | - | - | - | - |
| **CONT/OI** | 2.28x10^-4^ | - | - | - | - | - | - | - | - | - |
| **OI+OI/CV+OI** | 5.84x10^-9^ | 1.16x10^-5^ | 9.30x10^-11^ | 4.22x10^-3^ | 3.04x10^-11^ | 2.34x10^-9^ | 2.42x10^-5^ | 6.08x10^-4^ | 3.19x10^-6^ | 3.06x10^-6^ |
| **CONT/CV+OI** | 8.90x10^-9^ | 1.01x10^-5^ | 6.09x10^-11^ | 1.98x10^-4^ | 2.07x10^-11^ | 4.58x10^-9^ | 5.51x10^-5^ | 1.87x10^-3^ | 4.12x10^-6^ | 3.39x10^-6^ |
| **CONT/OI+OI** | - | - | - | - | - | - | - | - | - | - |

**Supplementary Table S7.** Correlation analysis tested between variables for all experimental groups. Pearson and Spearman-rank correlation test were used for data analysis.

| **Correlation** | **Lung lesion score** | **IgA antibodies** |
| --- | --- | --- |
| **IL-4** | - | - |
| **IL-8** | - | - |
| **IFN-γ** | - | -0.43^P^ |
| **TGF-β** | - | 0.95^P^ |
| **qPCR lungs** | 0.87^S^(CV)^1^, 1^S^(CV+OI)^1^ | - |
| **qPCR BALF** | 0.99^P^(CV)^1^, 0.97^P^(OI)^1^, 0.96^P^(CV+OI)^1^, 0.99^P^ (OI+OI)^2^ | - |
| **Histology** | - | - |
| **IgG** | - | Nt |
| **IgA** | - |  |

^1^28 days post-infection; ^2^56 days post-infection; ^S^Spearman correlation coefficient; ^P^Pearson correlation coefficient; (-) Absence of correlation; (Nt) Not tested. P-values < 0.05. The data involving lung lesion score showed significant differences between the vaccinated and control groups and were analyzed separately. The data involving IgA anti-*M. hyopneumoniae* and cytokines were tested for each group and also for the entire data set, since there was no significant difference between groups with regard to the expression of cytokines.
